# Development of a Novel Benzimidazole-Based Probe and Portable Fluorimeter for the Detection of Cysteine in Human Urine

**DOI:** 10.3390/bios11110420

**Published:** 2021-10-26

**Authors:** Gyu Seong Yeom, In-ho Song, Shrikant Dashrath Warkad, Pramod B. Shinde, Taewoon Kim, Seong-min Park, Satish Balasaheb Nimse

**Affiliations:** 1 Institute of Applied Chemistry and Department of Chemistry, Hallym University, Chuncheon 24252, Korea; m21019@hallym.ac.kr (G.S.Y.); songin-ho@hallym.ac.kr (I.-h.S.); psm3561@naver.com (S.-m.P.); 2 Biometrix Technology, Inc., 2-2 Bio Venture Plaza 56, Chuncheon 24232, Korea; shrikant@bmtchip.com; 3 Natural Products & Green Chemistry Division, CSIR—Central Salt and Marine Chemicals Research Institute (CSIR-CSMCRI), Council of Scientific and Industrial Research (CSIR), Bhavnagar 364002, Gujarat, India; pramodshinde@csmcri.res.in; 4 School of Software, Hallym University, Chuncheon 24252, Korea; taewoon@hallym.ac.kr

**Keywords:** cysteine, biothiols, cystinuria, portable, fluorimeter, bio-imaging, cancer

## Abstract

The measurement of cysteine in human urine and live cells is crucial for evaluating biological metabolism, monitoring and maintaining the immune system, preventing tissue/DNA damage caused by free radicals, preventing autoimmune diseases, and diagnosing disorders such as cystinuria and cancer. A method that uses a fluorescence turn-on probe and a portable fluorescence spectrometer device are crucial for highly sensitive, simple, rapid, and inexpensive cysteine detection. Herein, we present the synthesis and application of a benzimidazole-based fluorescent probe (**ABIA**) along with the design and development of a portable fluorescence spectrometer device (**CysDDev**) for detecting cysteine in simulated human urine. **ABIA** showed excellent selectivity and sensitivity in detecting cysteine over homocysteine, glutathione, and other amino acids with the response time of 1 min and demonstrated a detection limit of 16.3 nM using the developed **CysDDev**. Further, **ABIA** also demonstrated its utility in detecting intracellular cysteine, making it an excellent probe for bio-imaging assay.

## 1. Introduction

Biothiols such as cysteine (Cys), homocysteine (Hcy), and glutathione (GSH) play a vital role in numerous biological reactions [1,2,3,4,5,6]. It is important to note that among other biothiols, Cys deficiency exhibits several disorders, including edema, liver damage, muscle and fat loss, narcolepsy, skin damage, and weakness [7,8,9]. On the other hand, elevated cysteine levels are often correlated with cystinuria, a metabolic disorder characterized by the urinary loss of amino acids including Cys, arginine, ornithine, and lysine [10]. Therefore, measuring Cys in human urine and live cells is crucial for evaluating biological metabolism, monitoring and maintaining the immune system, preventing tissue/DNA damage caused by free radicals, preventing autoimmune diseases, and diagnosing disorders such as cystinuria and cancer.

There have been tremendous advances in developing methods for detecting biothiols in serum and live cells [11,12,13,14]. The development of fluorescent probes for detecting Cys has attracted significant attention from the broader scientific community [15,16,17,18]. The simplicity, high sensitivity and selectivity, and suitability for noninvasive and real-time detection are the significant advantages of methods based on fluorescent probes [19,20,21,22]. Though there are numerous reports on mechanistically different fluorescent probes for detecting biothiols [23,24,25,26], only a few have been applied to simultaneously detect Cys in human urine and live-cell imaging [27,28,29]. Therefore, using a fluorescent probe that can detect Cys in human urine and live cells can result in an excellent method for diagnosing cystinuria and Cys-related disorders [30].

There have been several reports on the colorimetric detection of Cys using paper-based devices [31,32]. However, proteins and large quantities of amino acids can interfere in color development reactions. The other disadvantage of paper-based devices is a higher limit of detection (LOD) [33,34]. Hence, paper-based devices can only provide qualitative information on urinary cysteine. Further, the fluorescence-based detection of Cys in biological fluids is advantageous over other methods due to its simple operating procedure, high sensitivity and selectivity for Cys in the presence of other proteins and amino acids, lower LOD, and the possibility of developing a portable device applicable in point-of-care settings.

Herein, we report on developing and applying a Cys specific fluorescent probe and the portable device for detecting cysteine in urine. The probe **ABIA** (2-(anthracen-9-yl)-5-methyl-1H-benzo[d]imidazole-acrylamide), an organic dye, was prepared in a two-step reaction and was evaluated as the target probe for biothiols. **ABIA** demonstrated excellent selectivity for Cys over other biothiols and amino acids, with a response time of a mere 1 min. The application of **ABIA** in live-cell imaging was evaluated by inverted fluorescence microscopy in a lung cancer cell line (A549), proving that **ABIA** as a suitable tool for tracking Cys in vitro. Its applicability in detecting Cys in live cells and simulated urine prompted us to develop a portable fluorimeter. The design and development of Cys detection device (**CYsDDev**) is also elaborated upon here. **ABIA** and **CysDDev** allow the detection of Cys in simulated urine, with a LOD of 16.3 nM.

## 2. Materials and Methods

### 2.1. Materials and Instrumentation

Reagents and required chemicals were procured either from Sigma-Aldrich (Seoul, Korea) or TCI (Seoul, Korea) and were used as received unless stated otherwise. The simulated urine (product code, 84 0679) was obtained from Biozoa Biological Supply Company (Seoul, Korea). The reactions were conducted under an inert atmosphere (argon gas). Completion of the reaction was monitored using thin-layer chromatography (TLC) by visualizing the plates under UV light (254 nm). DMSO-*d*6 was used as a solvent to characterize synthesized compounds by ^1^H- and ^13^C-NMR spectroscopy on a Jeol FT-NMR spectrometer (400 MHz; JEOL, Tokyo, Japan). The chemical shifts (δ) and the coupling constants (J) were reported as ppm and Hz, respectively. The Shimadzu UV-24500 (Shimadzu, Tokyo, Japan) spectrometer and Agilent Cary Eclipse fluorescence spectrophotometer (Agilent Technologies, Santa Clara, CA, USA) were used to record UV-visible spectra and fluorescence emission spectra, respectively. A JMS-700 MStation Mass Spectrometer (JEOL, Tokyo, Japan), a microplate Reader Spectramax Plus 384 (Molecular Devices, San Jose, CA, USA), and a confocal laser scanning microscope (Carl Zeiss LSM710, Osnabrück, Germany) were also used in the present study. The material for 3D printing and the electronic components required for the development of **CysDDev** were procured from Devicemart (Seoul, Korea) and Mouser Electronics (Seoul, Korea). A CUBICON Single Plus 320C (Jungwon, Korea) 3D printer was used to fabricate various parts and the body of **CysDDev**.

### 2.2. Synthesis of Compound **3** (2-(Anthracen-9-yl)-5-methyl-1H-benzo[d]imidazole)

A solution of anthracene-9-carbaldehyde (0.84 g, 4.07 mmol) in ethanol (10 mL) was slowly added dropwise to a solution of 4-methylbenzene-1,2-diamine (0.5 g, 4.09 mmol) in ethanol (20 mL) with a dropping funnel for 2 h. Then, the reaction mixture was continually stirred under air at room temperature until the completion of the reaction. The progress of the reaction was monitored by TLC using an ethyl acetate:hexanes (3:7) mixture. The precipitated product was filtered. The filter cake was washed with three aliquots of 10 mL ethanol followed by vacuum drying to obtain the final product: beige-colored powder. Yield: 1.01 g (80%). ^1^H-NMR (400 MHz, DMSO-*d*_6_) δ 12.88 (s, 1H), 8.84 (s, 1H), 8.21 (d, J = 8.4 Hz, 2H), 7.69 (d, J = 8.7 Hz, 2H), 7.61–7.55 (m, 3H), 7.55–7.48 (m, 3H), 7.39 (s, 1H), 2.50 (s, 3H). ^13^C-NMR (100 MHz, DMSO-*d*_6_) δ 130.68, 130.58, 128.74, 128.50, 126.80, 126.02, 125.65, 21.34; HR-EIMS^+^: *m/z* = 308.1311 (M)^+^ (Calcd for C_22_H_16_N_2_, 308.13).

### 2.3. Synthesis of Probe **ABIA** (1-(2-(Anthracen-9-yl)-5-methyl-1H-benzo[d]imidazol-1-yl)prop-2-en-1-one)

A solution of compound **3** (0.1 g, 0.32 mmol), triethylamine (0.18 mL, 1.29 mmol), and DMAP (0.004 g, 0.033 mmol) in anhydrous tetrahydrofuran (25 mL) was stirred at 0 °C under argon gas for 30 min. Then, prop-2-enoyl chloride (0.104 mL, 1.29 mmol) was slowly added to the reaction mixture and continued to be stirred until the completion of the reaction. The reaction progress was monitored by TLC using an ethyl acetate:hexanes (3:7) mixture. After the completion of the reaction (1 h), 100 mL of CH_2_Cl_2_ was added to the reaction mixture. Then, the reaction mixture was washed three times with the 150 mL aliquots of saturated Na_2_CO_3_ followed by a single wash of 150 mL brine. The separated organic layer was then dried over anhydrous MgSO_4_, and the solvent was evaporated under reduced pressure to obtain the crude product, which was then purified by column chromatography (SiO_2_, ethyl acetate/hexane = 1/20, *v*/*v*). The final compound was obtained as a light yellow solid. Yield 82 mg (74%). ^1^H-NMR (400 MHz, DMSO-*d*_6_) δ 8.92 (s, 1H), 8.23 (d, J = 8.0 Hz, 2H), 8.13–7.96 (m, 1H), 7.85–7.69 (m, 1H), 7.59 (dd, J = 8.3, 4.4 Hz, 2H), 7.54 (d, J = 3.2 Hz, 4H), 7.38 (t, J = 9.8 Hz, 1H), 5.99 (dd, J = 16.9, 3.6 Hz, 1H), 5.71 (ddd, J = 16.9, 10.5, 6.5 Hz, 1H), 5.27 (d, J = 10.9 Hz, 1H), 2.54 (d, J = 14.1 Hz, 3H). ^13^C NMR (100 MHz, DMSO-*d*_6_) δ 164.38, 164.20, 149.45, 148.87, 143.21, 141.05, 135.18, 134.53, 133.39, 132.52, 131.19, 130.71 (A), 130.68 (B), 130.53, 130.01, 128.82, 128.37, 128.25, 127.81, 126.65, 126.25, 125.86, 124.75 (A), 124.62 (B), 119.90 (A), 119.63 (B), 115.16 (A), 114.89 (B), 21.60 (A), 21.08 (B). ((A), (B) represents peak separation due to tautomerization of **ABIA**, details are shown in Supporting information Figure S7 and Table S1). HR-EIMS^+^: *m/z* = 362.1418 (M)^+^ (Calcd for C_25_H_18_N_2_O, 362.14).

### 2.4. UV–Vis and Fluorescence Spectroscopy

The double-distilled water and spectroscopic grade DMSO were used to prepare the stock and working solutions. The working solution of **ABIA** (10 µM) was prepared by diluting the stock solution of **ABIA** (1 mM, in DMSO) with a mixture of 90% DMSO:0.01 M HEPES buffer. The working solutions (1 mM) of amino acids were prepared by diluting the stock solutions (10 mM, in double-distilled) with 0.01 M HEPES buffer. The UV-visible absorption and emission spectra of **ABIA** (10 µM) dissolved in 90% DMSO:0.01 M HEPES buffer were recorded at room temperature (298 K) by adding the solution of various analytes such as glycine (Gly), alanine (Ala), serine (Ser), proline (Pro), valine (Val), threonine (Thr), isoleucine (Ile), leucine (Leu), asparagine (Asp), aspartic acid (Asn), glutamine (Gln), lysine (Lys), glutamic acid (Glu), methionine (Met), histidine (His), phenylalanine (Phe), arginine (Arg), tyrosine (Tyr), tryptophan (Typ), homocysteine (Hcy), glutathione (GSH), cysteine (Cys), bovine serum albumin (BSA), and other biologically relevant ions, including Cl^−^, Br^−^, NO_3_^−^, AcO^−^, SO_4_^2−^, PO_4_^3−^, Na^+^, Cs^+^, Ca^2+^, and Cu^2+^ to examine the selectivity of **ABIA**.

For sensitivity study, the titration experiments were accomplished through a stepwise addition of 1.15 equivalents of Cys (1 mM) to a solution of **ABIA** (10 µM). The absorbance intensity and emission intensity were recorded in the range of 200–600 nm and 350–650 nm, respectively, alongside a reagent blank. The effect of reaction time on the Cys detection was determined by tracing the fluorescence intensity of **ABIA** (10 µM) in the presence of five equivalent Cys (50 µM) for 0 to 60 min. The response of **ABIA** for reaction with Cys obeyed pseudo-first-order kinetics. Therefore, the rate constant was calculated according to the following equation (Equation (1)) [35]:

(1) $$\ln(\left(F_{max}-F\right)/F_{max})=-kt\;$$

where *F* is the fluorescence intensity at time *t*, *F_max_* is the fluorescence intensity after the reaction, and *k* is the rate constant. The fluorescence intensity was recorded at λ_ex_/λ_em_ = 368/470 nm alongside a reagent blank with the excitation and emission slits set to 5.0 nm.

### 2.5. Fabrication of **CysDDev**

The 3D models of various parts and the main case were created in Autodesk Fusion 360 software. The device’s overall size was 130 × 120 × 50 mm (W × D × H). **CysDDev** was assembled with several parts, including the cuvette holder, mounting holes for the LED and sensor, lid for the LED and sensor, the main case, and a top cap lid for the cuvette holder. The cuvette holder could hold a plastic or quartz cuvette with the dimensions of 12.5 × 12.5 × 45 mm dimension. The main body had a hole connecting the device to the computer. When completely assembled and when the top cap lid was mounted, there was no significant light entering the measuring device. Apart from the top cap lid for the cuvette holder, there were no moving components in the device, making it easy to fabricate and assemble. The **CysDDev** prototype was optimized for fused deposition modeling (FDM) 3D printing. All three parts of the design were printed separately with black thermoplastic filament to lower the reflectivity of the internal surfaces. The electronics parts of the **CYSDDev** consisted of 9 V battery, a sensor (Hamamatsu micro-spectrometer C12666MA, Japan) with the detection range λ = 340–850 nm), a UV LED (15 mA, λ = 370 nm), an Arduino Uno R3 microcontroller for device control and data processing, and a computer connector cable. The LED drew power from a 9 V battery, and the Arduino microcontroller received the energy from the connected computer. The measured fluorescence profile of a sample by **CysDDev** was automatically converted into a Microsoft Excel file (.csv) consisting of the information on the wavelength (nm) and respective fluorescence intensity (a.u.).

### 2.6. pH Effect on Cys Detection by **ABIA**

The effect of pH (pH = 3–10) on the Cys detection using **ABIA** was examined by fluorescence spectroscopy. The tetrabutylammonium hydroxide and perchloric acid were used to adjust the pH of simulated human urine.

### 2.7. Detection of Cys Using **ABIA** and **CysDDev** in Simulated Human Urine and Real Urine Sample

Usually, urine pH is slightly acidic, with typical values of 6.0 to 7.5. However, the range of urine pH is 4.5 to 8.0. Cys detection using **ABIA** and **CysDDev** was performed using simulated urine with a pH of 6.5. A standard curve was obtained by diluting a stock solution of Cys in analyte-free simulated human urine (0–150 µM). Each sample was serially diluted with the analyte-free urine to obtain solutions with concentrations in the detection range. The mean of ten fluorescence signal measurement values (SD in the range of 1.5–9.8%) for each calibration point was used to construct the standard curve. The LOD was estimated by applying the IUPAC recommended equation, LOD = 3σ/slope [36]. Where σ is the standard deviation of (*n* = 10) blank samples and the slope for the calibration curves.

A real urine sample obtained from a healthy volunteer was used for the spiking test and to validate the applicability of the **ABIA** and **CysDDev** in clinical samples. An amount of 5 μL urine from a healthy volunteer spiked with various Cys concentrations was added to the **ABIA** solution, and the fluorescence intensities were measured. Then, the percent recovery was measured using the standard curve.

### 2.8. Cell Culture, Cytotoxicity Assay, and Bio-Imaging

The cytotoxicity assay (MTT (3-(4,5-dimethylthiazol-2-yl)-2,5-diphenyltetrazolium bromide) assay) of **ABIA**, Cys, and Cys combined with the probe **ABIA** was performed using A549 cells (adenocarcinomic human alveolar basal epithelial cells), as reported earlier [37,38]. In brief, about 7000 A549 cells per well were seeded in 96-well plates and were allowed to incubate for 24 h. Then, the solution in the wells was replaced by media containing **ABIA**, Cys, and **ABIA** with Cys (1, 10, 25, and 50 µM) and was incubated for another 24 h. The dimethyl sulfoxide (DMSO) was used as a control. About 200 µL of a media containing MTT solution was added to each well and was allowed to incubate for four hours at 37 °C. Finally, the absorbance was recorded at 570 nm to determine the cell cytotoxicity. The experiment was repeated three times, and the results were presented with mean and standard deviation.

The bio-imaging experiments were performed by seeding the A549 cells on glass-bottom culture dishes and incubating them for 48 h. For control experiments, cells were only treated with the culture medium and were observed under the confocal microscope after being washed three times with 0.1 M PBS (0.5 mL). For treatment, cells were treated with 500 µM of Cys and 25 µM of **ABIA** separately and were allowed to incubate for 1 h at 37 °C and 5% CO_2_ and were then washed three times with 0.1 M PBS (0.5 mL) before observation. For the bio-imaging of Cys by **ABIA**, cells were incubated with 500 µM of Cys for 1 h followed by replacing the media containing 25 µM of **ABIA** and incubating the cells for another 1 h. Then, the cells were washed three times with 0.1 M PBS (0.5 mL) before observation. For another control experiment, cells were treated with 1 mM N-ethylmaleimide (NEM, a biothiol scavenger) for 30 min at 37 °C followed by washing three times with 0.1 M PBS (0.5 mL). Then, the cells were incubated in the presence of 25 µM of **ABIA** for 1 h followed being washed three times with 0.1 M PBS (0.5 mL) before observation. In a similar experiment, cells pre-tread with NEM (1 mM), as mentioned above, were treated with Cys (500 µM, for 1 h) followed being washed with 0.1 M PBS (0.5 mL × 3) and then incubated in the presence of **ABIA** (25 µM, 1 h) and finally being washed three times 0.1 M PBS (0.5 mL) before observation. Finally, the PBS (1 mL) was added to each glass-bottom culture dish, and then the fluorescence images were recorded using a confocal microscope (Carl Zeiss LSM710).

## 3. Results

### 3.1. Synthesis and Characterization of the Probe **ABIA**

**Scheme 1:**
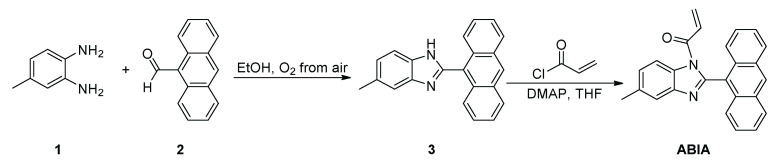
Scheme for the synthesis of the probe **ABIA**.

The probe **ABIA** was synthesized in a two-step reaction as depicted in Scheme 1.

In the first step, a simple yet efficient method for the synthesis of compound **3** was developed. The reaction of 4-methylbenzene-1,2-diamine (4.0 mmol) and anthracene-9-carbaldehyde (4.0 mmol) in ethanol at room temperature in the presence of O_2_ from the air and visible light allowed us to afford compound **3** with 80% yield (see the Supplemental Information Figures S1–S3). To the best of our knowledge, this is a first report on the synthesis of compound **3**. The reported synthetic procedures to obtain benzimidazole derivatives are different than the one employed here [39,40]. The previously reported methods used stringent reaction conditions, including dimethyl sulfoxide as a solvent, the temperature of 110 °C, and I_2_ as a catalyst. It is important to note that the procedure employed here uses mild reaction conditions, including ethanol as a solvent and 25 °C without using the additional catalyst. The method presented here allowed the synthesis of compound **3** with comparable yield.

**Figure 1:**
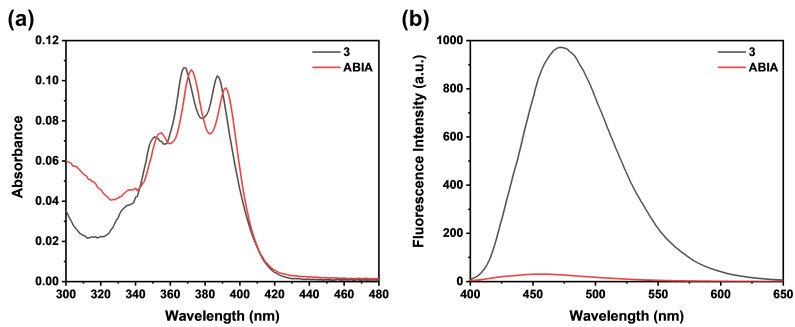
(**a**) UV-vis and (**b**) fluorescence emission spectra of compound **3** (10 µM; λ_ex_ = 368 nm) and the probe **ABIA** (10 µM) in 90% DMSO:0.01 M HEPES buffer solution (λ_ex_ = 372 nm).

In a second step, compound **3** was reacted with the prop-2-enoyl chloride (1.29 mmol) in the presence of triethylamine (1.29 mmol) and DMAP (0.033 mmol) to afford the final compound **ABIA** (see the Supplemental Information Figures S4–S6) in comparable yield. The ^1^H NMR and ^13^C NMR spectra of **ABIA** demonstrate the 5(6)-methyl tautomerism related to the N-substituted benzimidazoles (see the Supplemental Information Figure S7 and Table S1). According to previous reports, the tautomerism in benzimidazole derivatives is a well-known phenomenon, and in most cases, the separation of tautomers by column chromatography is not feasible [41,42]. Hence, we used the tautomeric mixture of **ABIA** in further experiments. The photophysical properties of compound **3** (10 µM) and probe **ABIA** (10 µM) in 90% DMSO:0.01 M HEPES buffer solution did not indicate a significant effect of tautomerism in **ABIA**. As shown in Figure 1, the optical absorbance spectrum of compound **3** shows λ_ex_ at 368 nm and another two peaks at 353 nm and 388 nm. The emission spectra of compound **3** show λ_em_ at 475 nm (λ_ex_ = 368 nm), indicating Stoke’s shift of 107 nm. Similarly, the probe **ABIA** demonstrates three distinct peaks (356, 372, and 392 nm) with the absorption maxima at 372 nm.

Interestingly, **ABIA** showed fluorescence (λ_em_ = 453 nm) that was almost 30-fold lower than compound **3**, indicating that the intramolecular charge transfer induced fluorescence quenching due to the N1-substituted acrylate group. The acrylate group has a strong electron-withdrawing ability that enhances the fluorescence turn-off effect in **ABIA**. These results indicate that reaction-induced removal of the acrylate group in **ABIA** can result in compound **3** with the fluorescence turn-on effect. Hence, the spectral characteristics of compound **3** were studied using various ratios (10, 50, 70, and 90%) of the DMSO:0.01 M HEPES buffer solution. As shown in Figure S8 and Table S2, compound **3** shows aggregation-induced fluorescence quenching with the increasing water percentage. It is important to note that 10% DMSO DMSO:0.01 M HEPES buffer solution completely dissolved 10 µM of compound **3**. However, compound **3** showed maximum fluorescence in 90% DMSO:0.01 M HEPES buffer solution. Hence, other fluorimetric experiments were conducted in 90% DMSO: 0.01 M HEPES buffer solution (λ_ex_ = 368 nm).

### 3.2. Determination of Selectivity of the Probe **ABIA** as a Chemosensor for Cys

**Figure 2:**
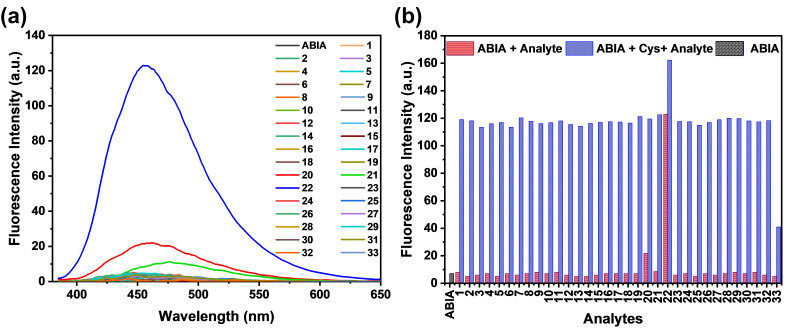
(**a**) Fluorescence spectra and (**b**) competitive study of **ABIA** (10 µM, λ_ex_ = 368 nm, λ_em_ = 455 nm) in the absence and presence of five equivalents of various analytes (50 µM). 1, Gly; 2, Ala; 3, Ser; 4, Pro; 5, Val; 6, Thr; 7, Ile; 8, Leu; 9, Asn; 10, Asp; 11, Gln; 12, Lys; 13, Glu; 14, Met; 15, His; 16, Phe; 17, Arg; 18, Tyr; 19, Typ; 20, Hcy; 21, Gsh; 22, Cys; 23, BSA; 24, Cl^−^; 25, Br^−^; 26, NO_3_^−^; 27, AcO^−^; 28, SO_4_^2−^; 29, PO_4_^3−^; 30, Na^+^; 31, Cs^+^; 32, Ca^2+^; 33, Cu^2+^.

The selectivity of the probe **ABIA** for Cys detection was investigated using fluorescence spectroscopy. The fluorescence spectra of **ABIA** (10 µM, in 90% DMSO:0.01 M HEPES buffer solution) were recorded immediately (1 min) after adding the five equivalents of various amino acids, BSA, anions (Cl^−^, Br^−^, NO_3_^−^, AcO^−^, SO_4_^2−^, PO_4_^3−^), and cations (Na^+^, Cs^+^, Ca^2+^, Cu^2+^) to the solution containing **ABIA**. As shown in Figure 2a, **ABIA** showed negligible fluorescence. However, the fluorescence intensity of **ABIA** increased significantly in the presence of Cys (λ_em_ = 455 nm) compared to the other analytes. Among the mercapto group-containing amino acids, **ABIA** showed 6-fold and 15-fold higher selectivity for Hcy (λ_em_ = 460 nm) and GSH (λ_em_ = 475 nm), respectively. In contrast, the selectivity for Cys was 50-fold higher than other amino acids. Further, **ABIA** did not show any interaction with biologically relevant ions such as Cl^−^, Br^−^, NO_3_^−^, AcO^−^, SO_4_^2−^, PO_4_^3−^, Na^+^, Cs^+^, Ca^2+^, and Cu^2+^. As shown in Figure 2b, in competition experiments conducted in the presence of an excess of (five equivalents) other amino acids, including Hcy and GSH, **ABIA** did not show any interference in the detecting Cys. However, among the tested biologically relevant ions, only Cu^2+^ affected the detection of Cys at tested equimolar concentrations (50 µM). Cys forms a coordination complex with Cu^2+^ ions using the mercapto and carboxylate functional groups [43]. Therefore, the complexation of Cys with Cu^2+^ inhibits its nucleophilic addition ability to the acrylate group of **ABIA**. However, the amount of Cu^2+^ (180 nM) [44] is negligible compared to Cys in healthy human urine (120 µM) and in the urine of cystinuria patients (2000 µM) [45,46]. Thus, Cu^2+^ would not interfere in Cys detection using the probe **ABIA** in the actual urine sample. These results indicate that **ABIA** is a highly selective fluorescence turn-on probe for Cys, and it can be applied to detect intracellular and urinary Cys.

To estimate the minimum response time, we traced the time-dependent fluorescence intensity of **ABIA** (10 µM) for 60 min upon the addition of five equivalents of Cys, as shown in Figure S9. The fluorescence intensity almost reached the maximum (310 a.u.) in 10 min after the addition of Cys. It is important to note that the fluorescence intensity was 121 a.u. within 1 min after adding Cys, which was comparatively fast compared to the reported probes, considering the structural simplicity of **ABIA** (see the Supplemental Information Table S3). In addition, we calculated the pseudo-first-order rate constants of the reactions between **ABIA** (10 µM) and five equivalents of Cys. The calculated pseudo-first-order rate constants for Cys from the linear regression analysis of time-dependent changes in the fluorescence intensity was 5.13 × 10^−3^ s^−1,^ as shown in Figure S9. These results indicate that the **ABIA** shows high specificity for Cys and a short reaction time (1 min). Hence, **ABIA** is applicable for the detection and quantification of Cys in urine and live cells.

**Figure 3:**
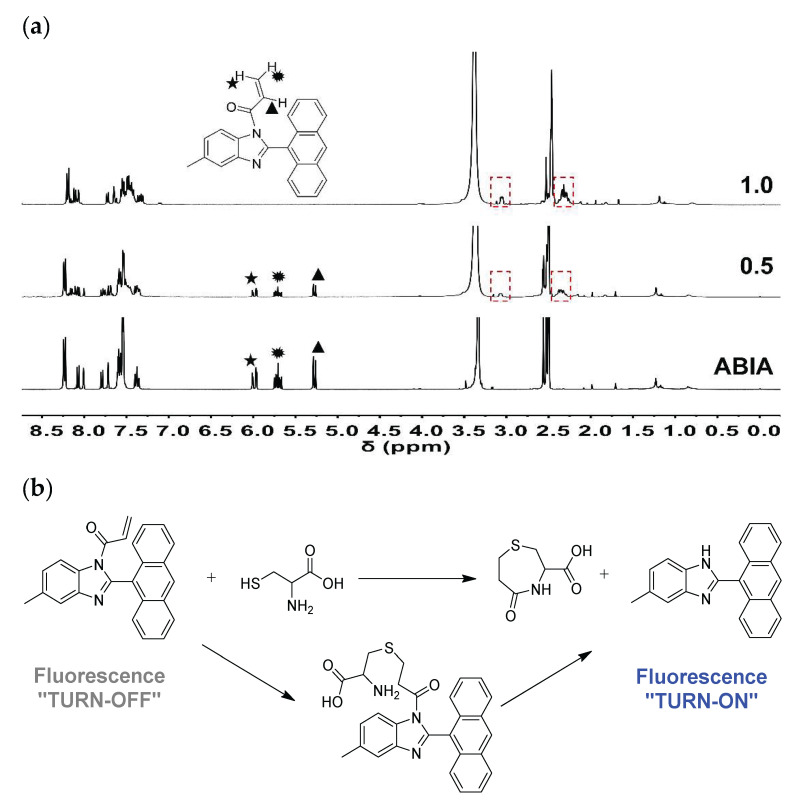
(**a**) Changes in ^1^H NMR spectra of **ABIA** upon the addition of 0.5 and 1.0 equivalents of Cys in 90% DMSO-*d*_6_:0.01 M HEPES buffer solution (D_2_O); (**b**) proposed Cys detection mechanism by **ABIA**.

The Cys detection mechanism by **ABIA** was studied using ^1^H NMR spectroscopy and mass spectroscopy. As shown in Figure 3, 0.5 and 1.0 equivalents of Cys were added to the 90% DMSO:0.01 M HEPES buffer solution (DMSO-*d*_6_ and D_2_O were used to make this solution) containing **ABIA**. Upon reaction, the peaks at 5.26, 5.71, and 5.97 ppm corresponding to the acrylate group of **ABIA** completely disappeared with 1.0 equivalents of Cys. These results indicate that the mercapto group in Cys undergoes the Michael addition with the double bond in the acrylate group of **ABIA.** Further, the appearance of new peaks at 2.26, 2.34, and 3.05 ppm indicates the intramolecular attack of the amine group of Cys on the carbonyl of **ABIA**, resulting in the cyclized product along with compound **3**, as shown in Figure 3b. The formation of compound **3** after the reaction of **ABIA** with Cys indicates the resultant fluorescence “turn-on” mechanism. Further proof for the proposed mechanism presented in Figure 3b was deduced from the mass spectra of a reaction product obtained by reacting equimolar **ABIA** and CYS for 60 min at 25 °C in 90% DMSO:0.01 M HEPES buffer solution. The solvent was evaporated at 40 °C under high-vacuum, and the resultant residue was prepared for the acquisition of the mass spectra by dissolving it in methanol. As presented in Figure 3b and Figure S10, the cyclization process of Cys after the reaction with **ABIA** generates a seven-membered ring (calcd. (H^+^): 176.0376; found *m/z*: 176.0) and compound **3** (calcd. (H^+^): 308.368; found *m/z*: 308.0) upon the de-acrylation of **ABIA**. Therefore, these results indicate the high applicability of **ABIA** as a fluorescence turn-on sensor for Cys in live cells and urine samples.

### 3.3. The Design and Application of **CysDDev** for Detecting Cys in Simulated Human Urine

**Figure 4:**
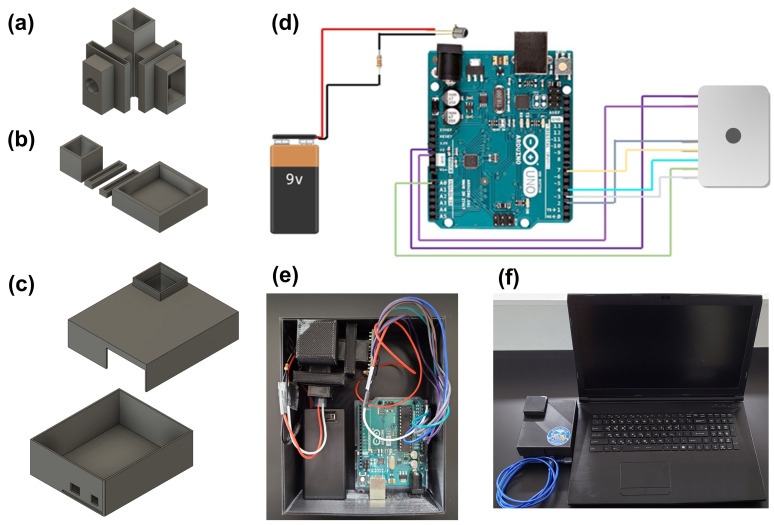
Schematic of various parts of the **CysDDev** (**a**): UV LED, sensor, and cuvette holder; (**b**) top lids for UV LED, sensor, cuvette holder; (**c**) the main case with top lid; (**d**) electronic components connected to the Arduino microcontroller; (**e**) assembly of **CysDDev** without the top lid; (**f**) **CysDDev** prototype connected to the laptop computer.

A schematic of the **CysDDev** prototype is shown in Figure 4. The LED was arranged in a nozzle opening at a 90° angle to the sensor nozzle in the cuvette holder, allowing efficient sample illumination by UV LED and detecting emitted light by the sensor. An electric current of 15 mA was delivered to the UV LED (λ = 370 nm) under a bias of 9 V using a rechargeable Li-ion battery. The analog signals from the sensor consisted of a train of pulses correlating to the spatial position of 256 pixels corresponding to wavelengths in the range of 340–850 nm. The analog data collected from the analog pin of the sensor were digitized in a bit resolution of 10 bits using the Arduino Uno R3 microcontroller that sends the processed data to the computer. The final data of individual sample readings were saved as a Microsoft Excel file (.csv) in the pre-selected folder on the connected computer.

**Figure 5:**
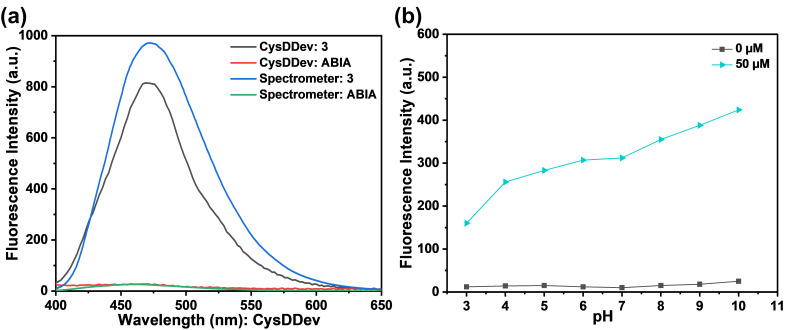
(**a**) Fluorescence spectra of compound **3** (10 µM, λ_ex_ = 370 nm) and **ABIA** (10 µM, λ_ex_ = 370 nm) recorded using **CysDDev** and commercial fluorescence spectrometer; (**b**) effect of pH on the detection of Cys (0, 50 µM) in simulated human urine at various pH using **ABIA** and **CysDDev** (λ_ex_ = 370 nm, λ_em_ = 455 nm).

We compared the performance of **CysDDev** with the commercial fluorescence spectrometer using the **ABIA** (10 µM) and compound **3** (10 µM) in 90% DMSO:0.01 M HEPES buffer solution. As shown in Figure 5a, the **CysDDev** showed comparable results with the commercial fluorescence spectrometer for compound **3** and the probe **ABIA**. These results indicate that the **CysDDev** developed here can detect Cys in various solutions, including in urine.

### 3.4. Effect of pH on the Detection of Cys by **ABIA**

The effect of pH (pH = 3–10) on the detection of Cys using **ABIA** was examined by using **CysDDev** both in the absence and presence of Cys (50 µM) in simulated human urine, as shown in Figure 5b. The fluorescence intensity of the probe **ABIA** in the absence of Cys did not show a significant change in the studied pH range of 3–10, indicating that the **ABIA** has remarkable stability in acidic and alkaline environments. The fluorescence intensity increased in the presence of various concentrations of Cys from pH 3.0–10.0. The *pKa* values of the Cys for α–NH_3_^+^ (*pKa* = 10.25) and –SH (*pKa* = 8.0) groups dictate that below pH 8, the –SH is the only nucleophile reacting with the acrylate group of **ABIA**. However, over pH 8, the nucleophilicity of Cys α–NH_2_ increases. Hence, over pH 8, the –SH and –NH_2_ groups of Cys can react with **ABIA** and can enhance the de-acrylation process, resulting in higher fluorescence intensity with increasing pH. Unlike reported probes for Cys detection that require use at pH ≥ 9.0 [47,48,49], **ABIA** can detect Cys in the physiological pH range and the typical urine pH values of 6.0 to 7.5.

### 3.5. Detection of Cys Using **ABIA** and **CysDDev** in Simulated Human Urine and Real Urine Sample

**Figure 6:**
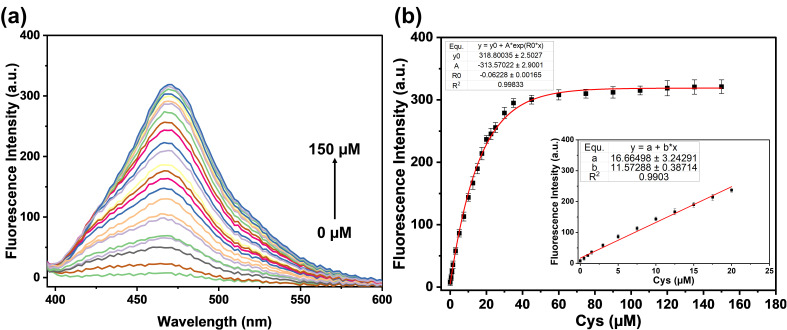
(**a**) Changes in fluorescence spectra of **ABIA** (10 µM, λ_ex_ = 370 nm) recorded using **CysDDev** upon the successive addition Cys (0–150 µM) in urine at pH = 7.3 (**b**) Calibration curves for the detection of Cys in urine using **ABIA** and **CysDDev** (λ_ex_ = 370 nm, λ_em_ = 455 nm) Inset is the linear regression curve in the range of 0–20 µM of Cys.

Cys detection using **ABIA** and **CysDDev** was performed using urine with a pH of 7.4. As shown in Figure 6, a calibration plot (0–150 µM) was obtained by diluting a stock solution of Cys.

As depicted in Figure 6, the fluorescence signals of **ABIA** (10 µM) increase linearly upon the successive addition of Cys until 20 µM and then reach the plateau around 50 µM and remains the same until 150 µM. The LOD for detecting Cys by **ABIA** using **CysDDev** was found to be 16.3 nM, with a linear detection range of 0–20 µM. It is imperative to note that **ABIA** demonstrated relatively lower detection limits for Cys than the other methods presented in Table S4.

Here, we used the standard addition method to quantify the Cys in the urine sample of a healthy volunteer to minimize the interference from the urine components. The fresh urine sample without any pretreatment was spiked with 3, 6, and 9 μM of Cys, and the spiked recovery was measured using the standard curve (Figure 6b (inset)). The results of the spike test are presented in Table 1. As shown in Table 1, the probe **ABIA** and **CysDDev** can effectively detect the Cys in the real urine sample with statistically significant recovery (99.20–104.9%) and precision (RSD < 1.45%).

### 3.6. Cell Imaging Application of **ABIA** for the Detection of Cys

**Figure 7:**
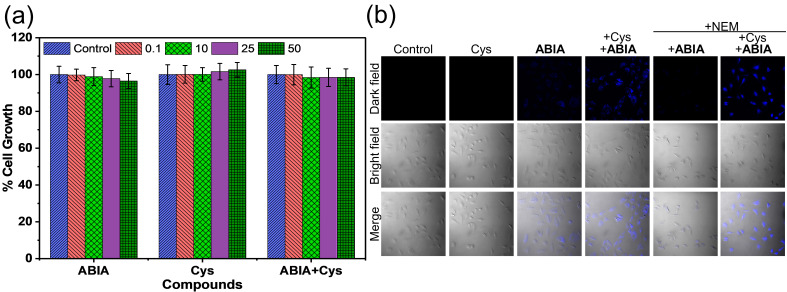
(**a**) Cytotoxicity’s of **ABIA**, Cys, and **ABIA** + Cys on A549 cells at concentrations of 0.1, 10, 25, and 50 µM after 24 h; (**b**) Dark field, bright field, and merged images upon treatment of A549 cells with control, Cys, **ABIA**, Cys + **ABIA**, NEM + **ABIA**, and NEM + Cys + **ABIA**.

The MTT assay allowed us to estimate cytotoxicity by treating cells with 0.1, 10, 25, and 50 µM of **ABIA**, Cys, and **ABIA** + Cys for 24 h using DMSO as a control. The cytotoxicity assay results are depicted in Figure 7a as the percent cell growth after treatment with **ABIA**, Cys, and **ABIA** + Cys compared to the control. There was no significant cell death even after 24 h of treatment at all tested concentrations. Therefore, 25 µM of **ABIA** was used for the cell imaging applications. As shown in Figure 7b, the control cells, cells treated Cys (500 µM), and cells pretreated with NEM (1 mM) followed by treatment with **ABIA** (25 µM) did not show significant fluorescence. However, the fluorescence intensity was observed upon the treatment of cells with Cys (500 µM) followed by **ABIA** (25 µM). Further, the fluorescence intensities were also observed in the cells pretreated with NEM (1 mM) followed sequentially by the treatment of Cys (500 µM) and **ABIA** (25 µM). These results indicate that the fluorescence turn-on probe **ABIA** has high potential in biological applications detecting intracellular Cys in the in vitro assays.

## 4. Discussion

There are several reports on the methods that are developed for detecting biothiols. Fluorescence turn-on sensors have attracted attention because of their high sensitivity, ease of operation, and fluorescence imaging in living cells. The Michael addition reaction [50], cyclization with aldehydes [51], conjugate addition-cyclization reaction [52], cleavage of sulfonamide, sulfonate esters [53], etc., reaction mechanisms have been reported for Cys detection among these sensors.

Most of the reported Cys detection probes are based on the coumarin scaffold with larger and more complicated structures [54,55]. Therefore, developing a synthetically accessible fluorescent probe with a simple design that shows high sensitivity and selectivity for detecting Cys can demonstrate several applications. Among the various fluorescent probes used for detecting biothiols, acrylate groups provide a fast response to the mercapto group and have a higher ability to quench fluorescence due to their strong electron-withdrawing effect [43,56,57]. Further, there are a few benzimidazole derivatives, such as fluorescent chemosensors [58,59]. However, in most cases, the benzimidazole moiety demonstrates fluorescence in the UV region (λ_em_ < 400 nm) [60], which is not suitable for live-cell imaging applications. The probe **ABIA** presented here contains an anthracene moiety, due to which it demonstrates fluorescence in the visible region, unlike other reported benzimidazole derivatives.

Compound **3**, which was presented in this manuscript, exhibited high fluorescence, upon which the subsequent reaction with acryloyl chloride resulted in the non-fluorescent **ABIA**. The directly linked acrylate group to the benzimidazole skeleton triggered an intramolecular charge transfer (ICT), resulting in a fluorescence turn-off effect. However, the Michael addition of the Cys mercapto group to the acrylate of **ABIA** restricted the ICT process and results in a fluorescence turn-on effect. **ABIA** shows selectivity for Cys over Hcy, probably because the rection of **ABIA** with Cys generates a seven-membered ring. In comparison, a reaction of **ABIA** with Hcy would produce an eight-membered ring. The strain in an eight-membered ring is much higher than that in a seven-membered ring; thus, a favorable seven-membered ring product forms easily. **ABIA** demonstrated high selectivity and comparatively fast response time for Cys detection (see the Supporting Information Table S3). The live-cell imaging study presented here provides evidence that **ABIA** has high applicability for in vitro bioimaging for Cys.

Reported probes were used for either the bioimaging or detection of Cys in serum samples. Only a few reports elaborate upon Cys detection in urine. However, the probe **ABIA** presented here was evaluated for its dual application for detecting Cys in live cells and urine. The **CysDDev** explained here allows the efficient detection of Cys in simulated human urine. The LOD for Cys detection by the probe **ABIA** using **CysDDev** was found to be of 16.3 nM. The **CysDDev** and **ABIA** demonstrated excellent clinical applicability in terms of LOD for Cys detection compared to reported methods (see the Supporting Information Table S4). Though the further evaluation of **CysDDev** for clinical application is required, these initial results indicate that **CysDDev** is an excellent portable tool that is deployable in resource-limited settings. The portability and the ease of use make **CysDDev** a perfect point-of-care device for evaluating clinical samples.

## 5. Conclusions

The novel fluorescence “off-on” probe **ABIA** to detect the mercapto-group containing amino acids was designed and synthesized. In addition, a portable device, **CysDDev**, was also designed and developed to measure the Cys in urine. The probe **ABIA** and **CysDDev** exhibited highly sensitive, simple, rapid, and inexpensive cysteine detection ability. The acrylate group in **ABIA** reacted with the mercapto group in Cys by means of the Michael addition reaction mechanism. The Michael addition reaction was followed by selective intramolecular nucleophilic substitution, ensuring selective detection of Cys over Hcy and GSH. The response time of 1 min and a detection limit of 16.3 nM signifies the high clinical applicability of **ABIA** and **CysDDev**. Further, **ABIA** also demonstrated its utility in detecting intracellular cysteine, making it an excellent probe for bio-imaging assay.

These results suggest that the **ABIA** and **CysDDev** will provide new insights into developing convenient approaches in Cys and other biomarker detections through simple, accurate, and portable health products for potential biological applications.

## Figures and Tables

**Table 1:** Spike recovery test using real urine sample from a healthy volunteer.

Spiked Cys	Found Cys	Recovery	RSD (*n* = 3)
(μM)	(μM)	(%)	(%)
3	3.14	104.9	1.45
6	5.95	99.2	1.22
9	9.77	108.6	0.50

RSD, relative standard deviation.

## Data Availability

The data presented in this study are available upon request from the corresponding author.

## Supplementary Materials

The following are available online at https://www.mdpi.com/article/10.3390/bios11110420/s1, Figure S1: ^1^H-NMR spectrum of compound 3, Figure S2: ^13^C-NMR spectrum of compound 3, Figure S3: HR-mass spectrum of compound 3, Figure S4: ^1^H-NMR spectrum of ABIA, Figure S5: ^13^C-NMR spectrum of ABIA, Figure S6: HR-mass spectrum of ABIA, Figure S7: Structures of the probe ABIA tautomer’s A and B, Figure S8: (a) UV-vis absorbance spectra, (b) Fluorescence spectra of compound 3 in 10, 50, 70, and 90% DMSO:0.01 M HEPES buffer solution (λ_ex_ = 368 nm), Figure S9: (a), (b)Time-dependent fluorescence response and (c) pseudo-first-order kinetic plots of ABIA (10 µM) reaction with Cys (50 µM) in 90% DMSO:0.01 M HEPES buffer solution (λ_ex_ = 368 nm, λ_em_ = 455 nm), Figure S10: Mass spectra of a reaction product obtained by reacting equimolar ABIA and Cys for 60 min at 25 °C, Table S1: ^1^H NMR and ^13^C NMR chemical shifts (δ ppm) of the ABIA tautomer’s A and B, Table S1. ^1^H NMR and ^13^C NMR chemical shifts (δ ppm) of the ABIA tautomer’s A and B; Table S2: Photophysical properties of compound 3 (10 µM) in varying percentages of DMSO:0.01 M HEPES buffer solution, Table S3: A comparison of response time for cysteine detection by various methods, Table S4: A comparison of different probes used for cysteine detection.
