# Supplementary material for: Development of a Novel Benzimidazole-Based Probe and Portable Fluorimeter for the Detection of Cysteine in Human Urine
Source: Biosensors (Basel). 2021 Oct 26;11(11):420. doi: 10.3390/bios11110420 (PMC8615561; doi:10.3390/bios11110420)
Supplement: Supplementary file 1 [file biosensors-11-00420-s001.zip › biosensors-1397543-supplementary.pdf]

biosensors

MDPI

Supplementary Information

Development of a Novel Benzimidazole-Based Probe and Portable Fluorimeter for the Detection of Cysteine in Human Urine

Gyu Seong Yeom [1,†], In-ho Song [1,†], Shrikant Dashrath Warkad [2], Pramod B. Shinde [3], Taewoon Kim [4], Seong-min Park [1] and Satish Balasaheb Nimse [1,*]

[1] Institute of Applied Chemistry and Department of Chemistry, Hallym University, Chuncheon 24252, Korea; m21019@hallym.ac.kr (G.S.Y.); songin-ho@hallym.ac.kr (I.-h.S.); psm3561@naver.com (S.-m.P.)
[2] Biometrix Technology, Inc., 2-2 Bio Venture Plaza 56, Chuncheon 24232, Korea; shrikant@bmtchip.com
[3] Natural Products & Green Chemistry Division, CSIR—Central Salt and Marine Chemicals Research Institute (CSIR-CSMCRI), Council of Scientific and Industrial Research (CSIR), Bhavnagar 364002, Gujarat, India; pramodshinde@csmcri.res.in
[4] School of Software, Hallym University, Chuncheon 24252, Korea; taewoon@hallym.ac.kr
* Correspondence: satish_nimse@hallym.ac.kr
† These authors contributed equally to this work.

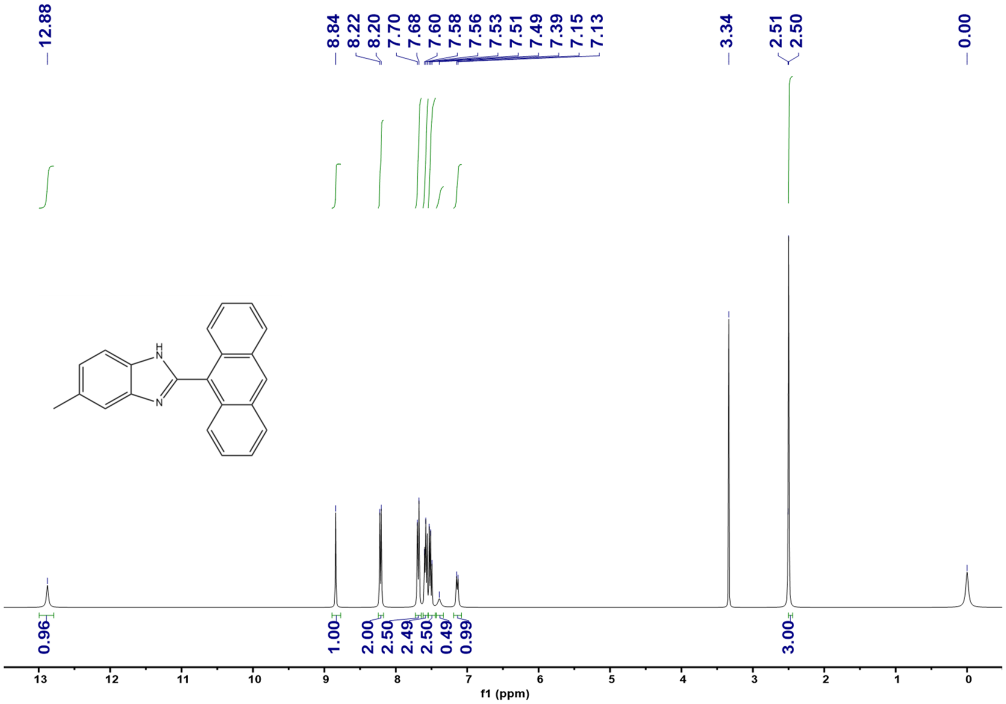

Figure S1. ^{1}H-NMR spectrum of compound **3**.

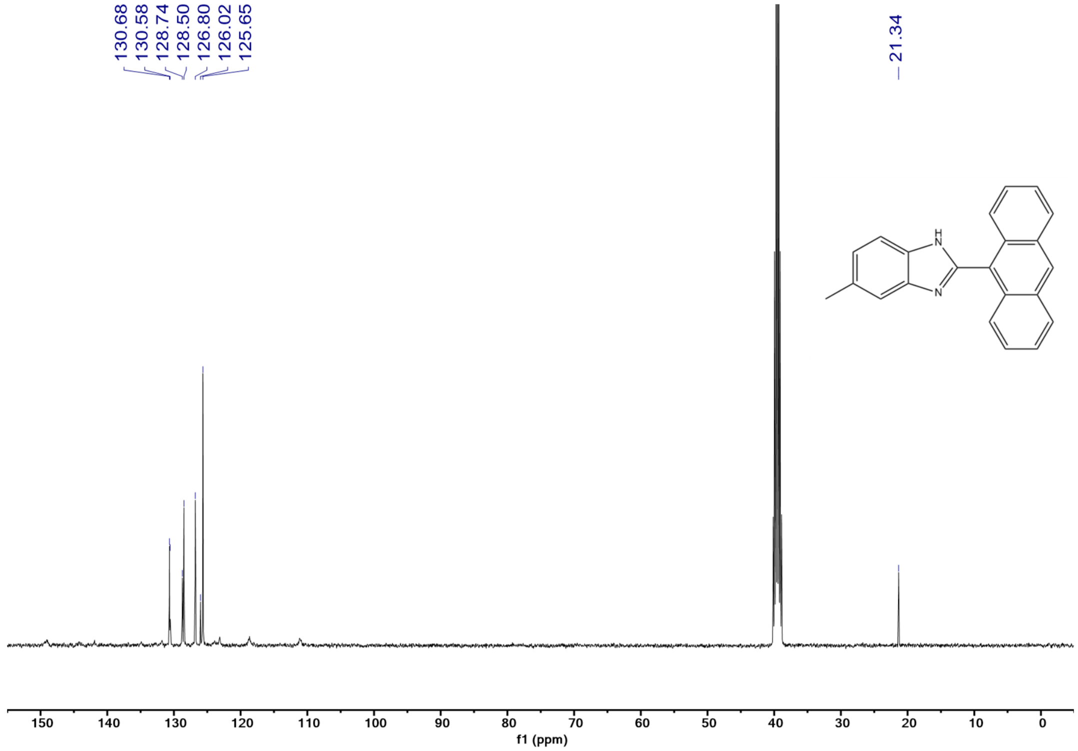

Figure S2. ^{13}C NMR spectrum of compound **3**.

[Mass Spectrum]
Data : B HR Date : 24-Aug-2021 16:06
RT : 1.04 min Scan# : 28
Elements : C 22/20, H 45/16, N 2/0
Mass Tolerance : 1000ppm, 5mmu if m/z < 5, 50mmu if m/z > 50
Unsaturation (U.S.) : -0.5 - 20.0

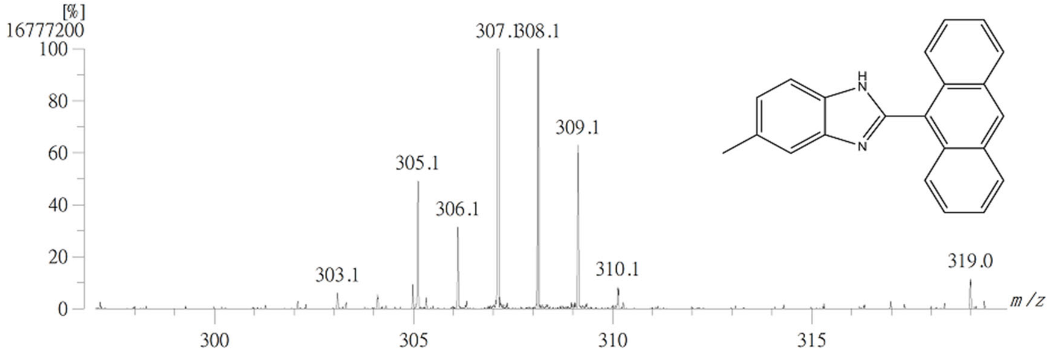

	Observed m/z	Int%	Err[ppm / mmu]	U.S.	Composition
1	308.1311	100.00	-0.8 / -0.2	16.0	C22 H16 N2

Figure S3. HR-mass spectrum of compound **3**.

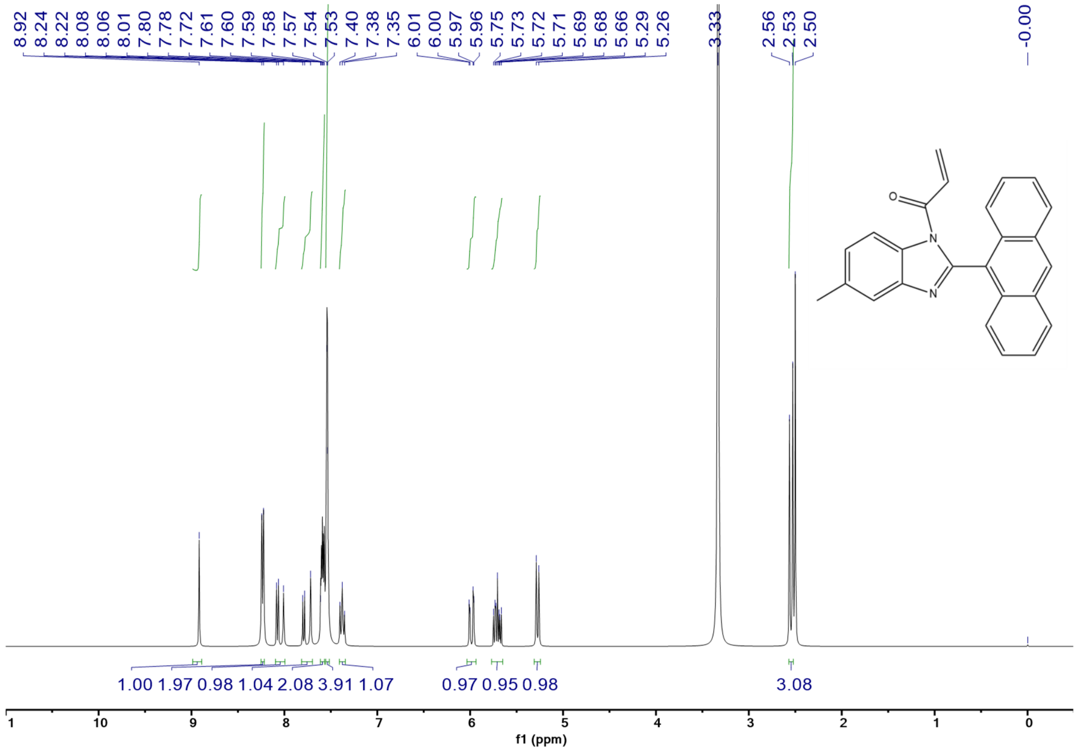

Figure S4. ^{1}H-NMR spectrum of **ABIA.**

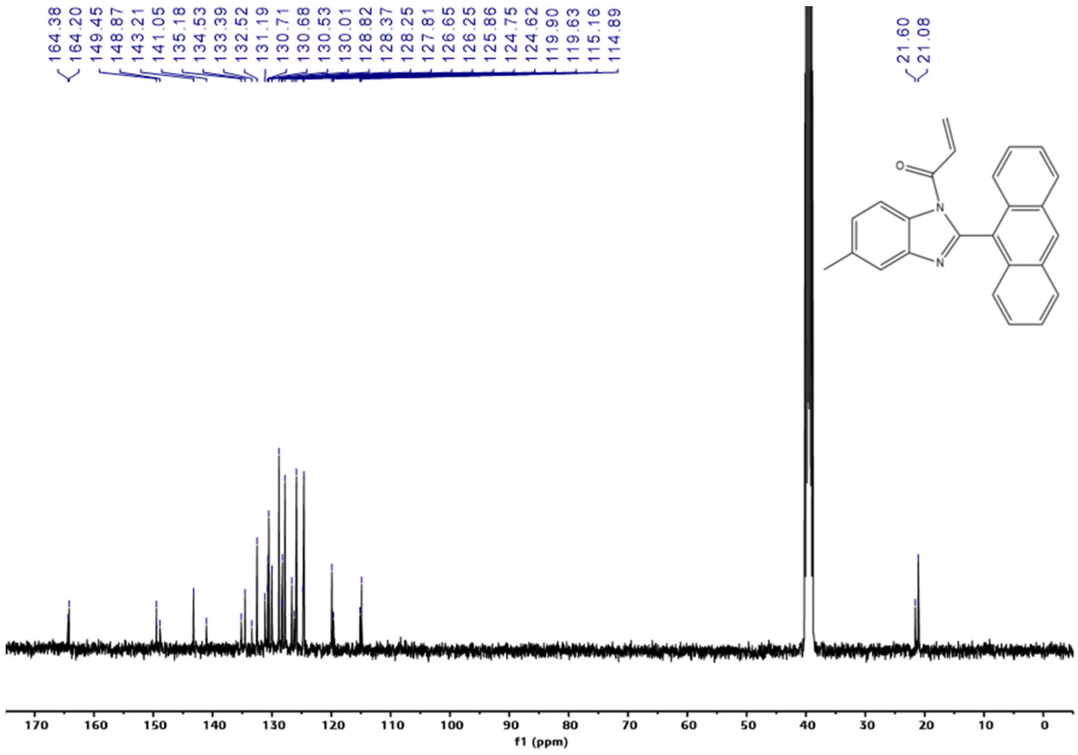

Figure S5. ^{13}C NMR spectrum of **ABIA.**

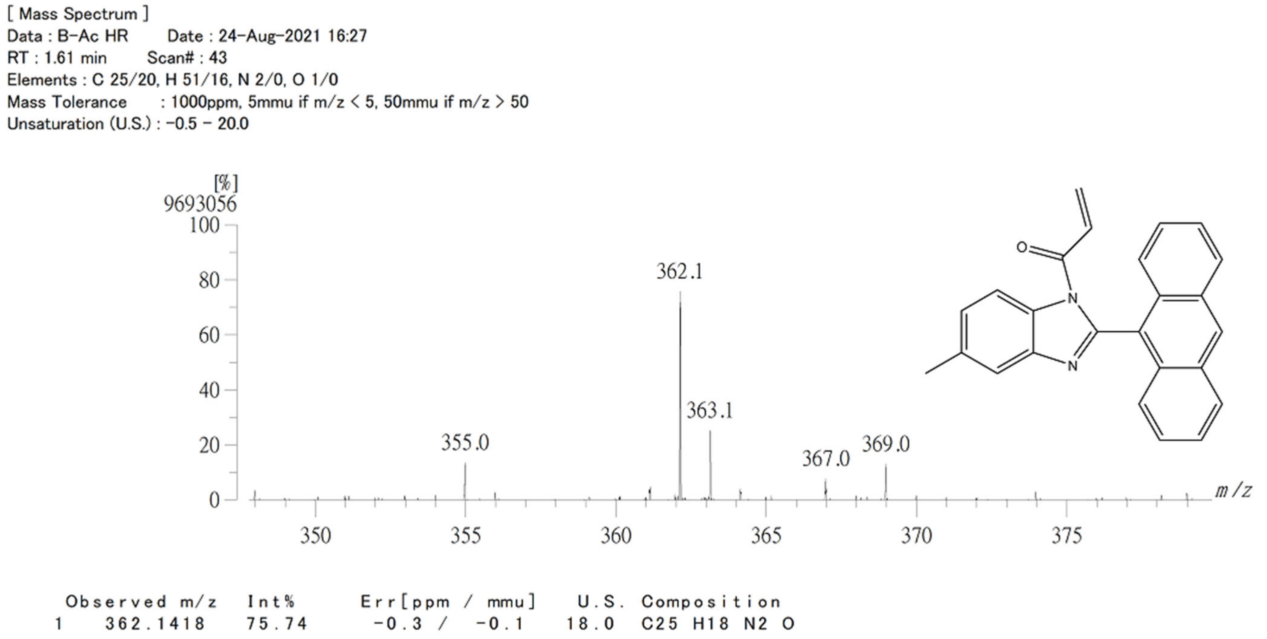

Figure S6. HR-mass spectrum of receptor **ABIA**.

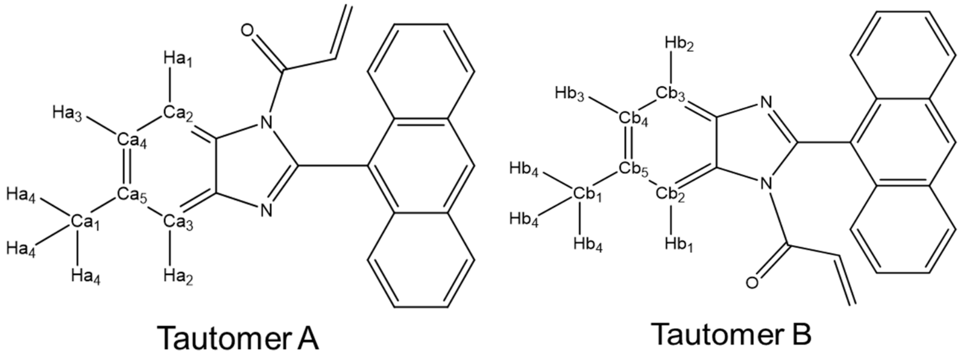

Figure S7. Structures of the probe **ABIA** tautomer's **A** and **B.**

Table S1. ^{1}H NMR and ^{13}C NMR chemical shifts (δ ppm) of the **ABIA** tautomer's **A** and **B.**

**Compound**	**ABIA**			
	**^{1}H NMR**		**^{13}C NMR**	
Proton or Caron Label	Tautomer A	Tautomer B	Tautomer A	Tautomer B
H[a,b]$_1$ / C[a,b]$_1$	8.06	7.80	130.71	130.68
H[a,b]$_2$ / C[a,b]$_2$	8.01	7.72	124.75	124.62
H[a,b]$_3$ / C[a,b]$_3$	7.40	7.35	119.90	119.63
H[a,b]$_4$ / C[a,b]$_4$	2.56	2.53	115.16	114.89
C[a,b]$_5$	-	-	21.60	21.08

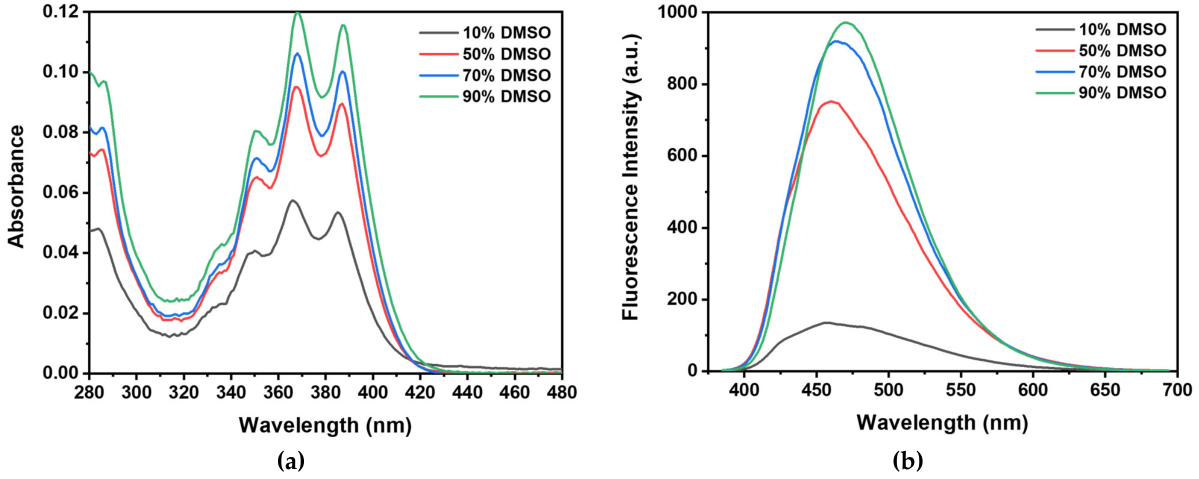

Figure S8. (**a**) UV-vis absorbance spectra, (**b**) Fluorescence spectra of compound **3** in 10, 50, 70, and 90% DMSO:0.01 M HEPES buffer solution (λ_{ex} = 368 nm).

Table S2. Photophysical properties of compound **3** (10×10^{-6} M) in varying percentages of DMSO:0.01 M HEPES buffer solution.

DMSO Percentage (%)	λ_{max} (nm)	ε 10^4 [M^{-1} cm^{-1}]	λ_{em} (nm)	Stoke's shift (nm)
**10%**	368	0.56	458	90
**50%**	368	0.95	460	92
**70%**	368	1.06	462	94
**90%**	368	1.20	470	102

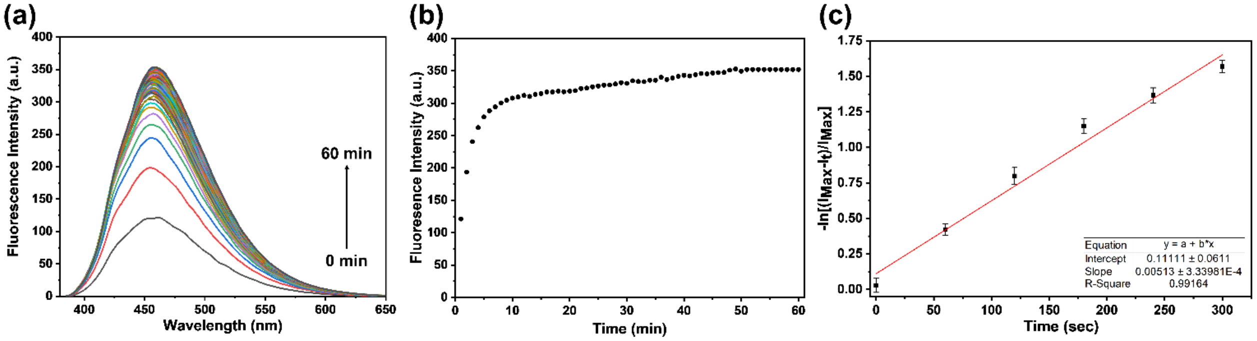

Figure S9. (**a**), (**b**)Time-dependent fluorescence response and (**c**) pseudo-first-order kinetic plots of **ABIA** (10×10^{-6} M) reaction with Cys (50×10^{-6} M) in 90% DMSO:0.01 M HEPES buffer solution (λ_{ex} = 368 nm, λ_{em} = 455 nm).

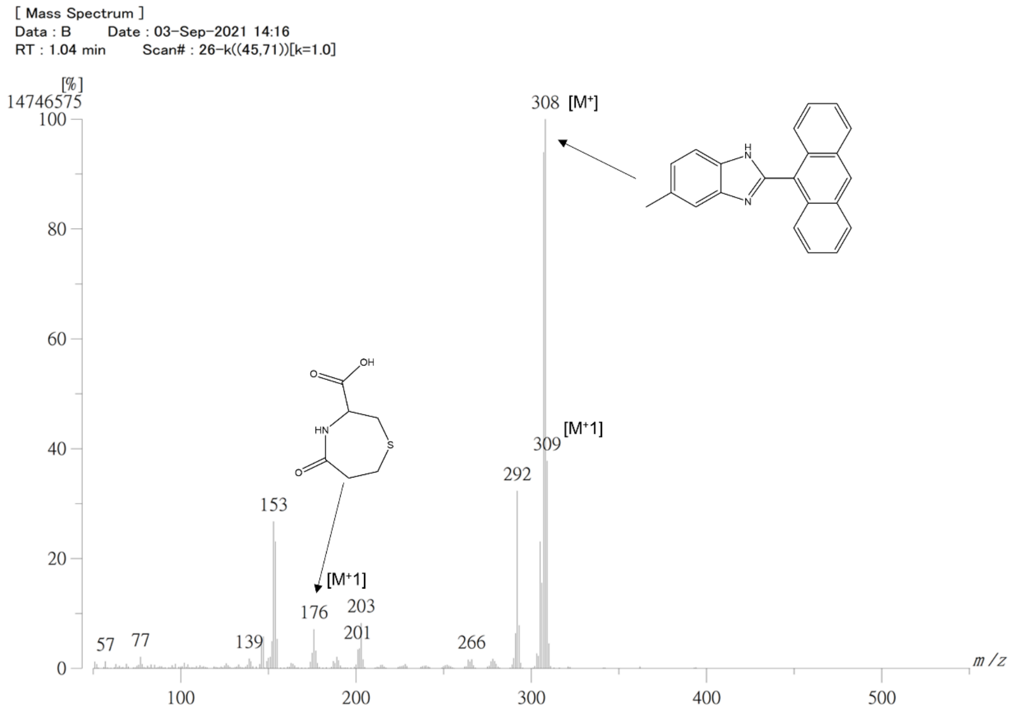

Figure S10. Mass spectra of a reaction product obtained by reacting equimolar **ABIA** and Cys for 60 min at 25 °C.

Table S3. A comparison of response time for cysteine detection by various methods.

Sr. No.	Probe	Response Time (min)	Rate Constant (s^{-1})	Ref.
**1**	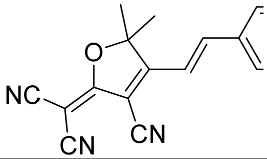	90	8.16×10^{-4}	[1]
**2**	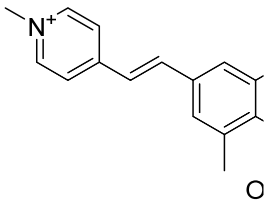	80	7.16×10^{-4}	[2]
**3**	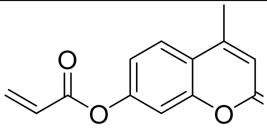	30	2.70×10^{-3}	[3]
**4**	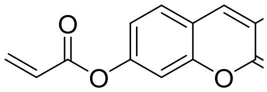	20	1.1×10^{-2}	[4]

**5**	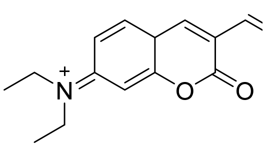	15	2.3 × 10^{-3}	[5]
**ABIA**	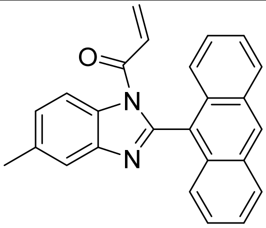	10	5.13 × 10^{-3}	**This Work**

Table S4. A comparison of different probes used for cysteine detection.

Sr. No.	Probe	LOD (nM)	Urine Test	Live Cell Imaging	Ref.
**1**	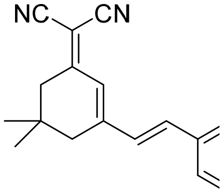	260	No	Yes	[6]
**2**	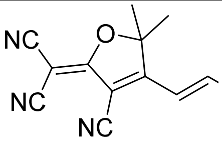	40	No	Yes	[7]
**3**	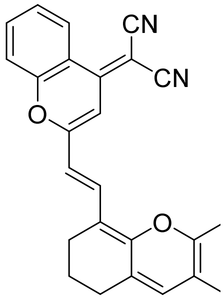	48	No	Yes	[8]
**4**	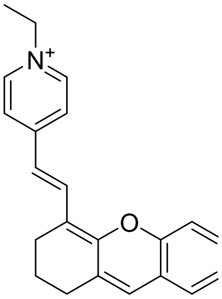	48.9	Yes	Yes	[9]

**5**	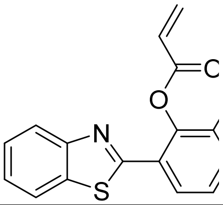	110	No	Yes	[10]
**ABIA**	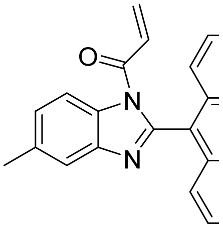	16.3	Yes	Yes	**This Work**

[a]–not reported.

References

1. Yang, S.; Zeng, Q.; Guo, Q.; Chen, S.; Liu, H.; Liu, M.; McMahon, M.T.; Zhou, X. Detection and differentiation of Cys, Hcy and GSH mixtures by (19)F NMR probe. *Talanta* **2018**, *184*, 513–519, doi:10.1016/j.talanta.2018.03.039.
2. Tang, L.; Xu, D.; Tian, M.; Yan, X. A mitochondria-targetable far-red emissive fluorescence probe for highly selective detection of cysteine with a large Stokes shift. *J. Lumin.* **2019**, *208*, 502–508, doi:10.1016/j.jlumin.2019.01.022.
3. Zeng, R.F.; Lan, J.S.; Li, X.D.; Liang, H.F.; Liao, Y.; Lu, Y.J.; Zhang, T.; Ding, Y. A Fluorescent Coumarin-Based Probe for the Fast Detection of Cysteine with Live Cell Application. *Molecules* **2017**, *22*, 1618, doi:10.3390/molecules22101618.
4. Qiao, H.; Meng, Y.; Zhang, Y.; Sun, J.; Wang, T.; Zhang, X.; Wang, F.; Kang, Y.-F. A simple coumarin-based fluorescent probe for specific detection of cysteine over homocysteine and glutathione. *Chem. Pap.* **2018**, *72*, 1461–1466, doi:10.1007/s11696-018-0401-2.
5. A, A.H.; Ali, F.; Kushwaha, S.; Taye, N.; Chattopadhyay, S.; Das, A. A Cysteine-Specific Fluorescent Switch for Monitoring Oxidative Stress and Quantification of Aminoacylase-1 in Blood Serum. *Anal. Chem.* **2016**, *88*, 12161–12168, doi:10.1021/acs.analchem.6b03066.
6. Zhang, W.; Liu, J.; Yu, Y.; Han, Q.; Cheng, T.; Shen, J.; Wang, B.; Jiang, Y. A novel near-infrared fluorescent probe for highly selective detection of cysteine and its application in living cells. *Talanta* **2018**, *185*, 477–482. doi: 10.1016/j.talanta.2018.04.001.
7. Zhang, B.; Zhang, H.; Zhong, M.; Wang, S.; Xu, Q.; Cho, D.-H.; Qiu, H. A novel off-on fluorescent probe for specific detection and imaging of cysteine in live cells and in vivo. *Chin. Chem. Let.* **2020**, *31*, 133–135, 10.1016/j.cclet.2019.05.061.
8. Qi, Y.; Huang, Y.; Li, B.; Zeng, F.; Wu, S. Real-Time Monitoring of Endogenous Cysteine Levels In Vivo by near-Infrared Turn-on Fluorescent Probe with Large Stokes Shift. *Anal Chem.* **2018**, *90*, 1014–1020. doi: 10.1021/acs.analchem.7b04407.
9. Dai, Y.; Zheng, Y.; Xue, T.; He, F.; Ji, H.; Qi Z. A novel fluorescent probe for rapidly detection cysteine in cystinuria urine, living cancer/normal cells and BALB/c nude mice. *Spectrochim Acta A Mol. Biomol. Spectrosc.* **2020**, *225*, 117490. doi: 10.1016/j.saa.2019.117490.
10. Yang, X.; Guo, Y.; Strongin, R.M. Conjugate addition/cyclization sequence enables selective and simultaneous fluorescence detection of cysteine and homocysteine. *Angew. Chem. Int. Ed. Engl.* **2011**, *50*, 10690–10693.
